# Interaction Between the *UCP2* rs659366 Polymorphism and Dietary Capsaicin Intake in Relation to the Inflammatory State in Mexican Adults

**DOI:** 10.3390/ijms262110419

**Published:** 2025-10-27

**Authors:** Ana Alondra Sobrevilla-Navarro, Bertha Landeros-Sanchez, Jose Roman Chavez-Mendez, Genaro Rodriguez-Uribe, Omar Ramos-Lopez

**Affiliations:** 1 Medicine and Psychology School, Autonomous University of Baja California, Tijuana 22390, Mexico; ana.sobrevilla@uabc.edu.mx (A.A.S.-N.);; 2 Faculty of Chemical Sciences and Engineering, Autonomous University of Baja California, Tijuana 22390, Mexico; 3 Health Sciences School “Valle de las Palmas”, Autonomous University of Baja California, Tijuana 22260, Mexico; roman.chavez@uabc.edu.mx

**Keywords:** *UCP2*, polymorphism, capsaicin, obesity, inflammatory state, personalised nutrition

## Abstract

Metabolic diseases such as obesity and related conditions have an inflammatory basis. Genetic and nutritional factors can influence the development of these diseases by altering the inflammatory state. This study aimed to analyse how the rs659366 (G/A) polymorphism in the *UCP2* gene interacts with dietary capsaicin (CAP) consumption and affects inflammatory markers in Mexican adults. A cross-sectional, analytical study was conducted in 212 adult patients. The *UCP2* rs659366 polymorphism was genotyped using an allelic discrimination assay. Dietary CAP intake was measured with a validated food frequency questionnaire. Multivariate linear regression analyses were performed for interaction analyses. The ancestral allele G accounted for 40.2% and the risk allele A accounted for 59.8% of samples. There was a significant interaction between CAP intake and the *UCP2* rs659366 polymorphism for the inflammatory marker NLR (neutrophil-to-lymphocyte ratio) (*p* < 0.05). Among subjects with the G allele, higher CAP intake was associated with higher NLR scores (*p* < 0.001). Patients with the G allele of the *UCP2* rs659366 polymorphism experienced increased inflammation with higher CAP intake. This finding highlights the need for future studies in personalised nutrition and could expand knowledge about the effects of CAP on obesity and inflammation.

## 1. Introduction

Metabolic diseases such as obesity and their associated comorbidities have an inflammatory basis. Inflammation is a rapid and controlled physiological process [1] triggered by both harmful external stimuli and infected or damaged host tissue [2]. If this inflammation is not properly coordinated and the condition persists, it can lead to pathological consequences, contributing to the development and progression of numerous diseases, such as type 2 diabetes, cardiovascular diseases, cancer, fatty liver disease, and other immune diseases. These inflammatory diseases are global health problems [3,4].

Obesity is a condition characterised by excessive body weight due to the abnormal accumulation of adipose tissue, involving genetic, metabolic, and environmental factors. It is a chronic condition that affects health by increasing the risk of other chronic diseases and reducing quality of life [5]. Recent studies have associated obesity with molecular-level factors such as localised inflammation in intestinal or adipose tissue and systemic inflammatory responses [6]. Therefore, understanding the basic cellular and molecular mechanisms is important to identify new therapeutic targets.

Natural products, such as bioactives derived from certain plants, have been evaluated for the treatment of various diseases, including inflammatory disorders [7]. Chili pepper (*Capsicum* spp.) is a traditional and widely consumed food in America, including Mexico [8]. Chili pepper produces a sensation of heat when consumed, commonly called “pungency”. Capsaicin (trans-8-Methyl-N-vanillyl-6-nonenamide, CAP) is the main chemical component of hot chili peppers. Studies have shown that CAP has anti-inflammatory, analgesic, antioxidant, and anticancer properties [9]. An analysis of CAP content using HPLC in hot chilies showed an average CAP concentration of 4249.0 ± 190.3 µg/g, with a pungency level of 67,984.60 Scoville heat units (SHU), where green peppers had the lowest detected concentration of 1.0 ± 0.9 µg/g, while red and yellow peppers were non-pungent [10].

The CAP receptor is the transient receptor potential vanilloid subfamily member 1 (TRPV1) vanilloid receptor, which may exert effects through a pathway independent of vanilloid receptors [11]. Previous studies show that CAP intake can improve vascular oxidative stress in diabetic mice by activating TRPV1, increasing protein kinase A (PKA) phosphorylation, and upregulating the uncoupling protein 2 (UCP2) protein [12].

Various genetic and nutritional factors may be associated with the onset and progression of different diseases and can affect the inflammatory state. Among the most studied genetic factors associated with inflammation is *UCP2*, a member of the mitochondrial anion carrier protein family, expressed in white adipose tissue and skeletal muscle [13,14].

CAP has demonstrated anti-obesity effects, which may be related to the activation of UCP proteins and the sympathetic nervous system. These properties induce metabolic activity by increasing thermogenesis. In a study involving 40 participants of both sexes, researchers investigated whether capsinoids could help reduce body fat and improve metabolism. Participants received 6 mg/day of oral CAP for 12 weeks, which was associated with a reduction in abdominal fat. Additionally, the response varied according to the presence of certain genetic polymorphisms, such as *TRPV1* Val585Ile and *UCP2* − 866 G/A, which may predict a therapeutic response [15]. The ingestion of CAP was significantly associated with an increase in fat oxidation, suggesting that UCP2 could be a predictor of therapeutic response [15]. Thus, UCP2 might play a central role as an etiological factor in the development of obesity and inflammatory diseases [16].

Some studies suggest that CAP may influence the expression of the *UCP2* gene, which could modulate the inflammatory response [17,18]. However, the relationship between genetic variants associated with CAP consumption, inflammation, and interaction with the *UCP2* gene in Mexican patients has apparently not been analysed. Therefore, a comprehensive study of these factors could help predict individual risk of inflammation and metabolic alterations and support the development of personalised nutrition strategies. The objective of this study was to analyse the interaction between the rs659366 (G/A) polymorphism in the *UCP2* gene and dietary CAP consumption in relation to inflammatory markers in an adult Mexican population.

## 2. Results

### 2.1. Distribution of the UCP2 rs659366 Polymorphism and Characteristics of the Studied Population

The allele frequencies of the *UCP2* rs659366 polymorphism were 40.2% for the ancestral allele G and 59.8% for the risk allele A. The genotypic frequencies were homozygous GG (3.7%), heterozygous GA (73%), and homozygous AA (23.3%). The characteristics of the studied population by *UCP2* genotypes are presented in Table 1. Sixty-seven percent of the patients were overweight or obese according to BMI. For the risk allele A, a greater HC (*p* = 0.040) was found compared to the ancestral allele G. The variables age, sex, and blood pressure were not significant among the *UCP2* genotypes.

### 2.2. Capsaicin Intake According to Genotypes of the UCP2 rs659366 Polymorphism

Table 2 presents the nutritional characteristics according to CAP consumption and *UCP2* alleles. No significant differences in CAP, appetite, total calories, or the percentage of proteins, fats, and carbohydrates were observed by *UCP2* alleles.

### 2.3. Biochemical Profile by Genotypes of the UCP2 rs659366 Polymorphism

Table 3 shows the biochemical profile of the patients according to *UCP2* alleles. Higher concentrations of glucose (*p* = 0.020), creatinine (*p* = 0.006), and creatine kinase (*p* = 0.035) were found in patients with the risk allele A, while higher concentrations of total protein markers (*p* = 0.039) and total bilirubin (*p* = 0.001) were detected in those with the ancestral allele G. No significant differences were found in the lipid profile or other biochemical markers analysed.

### 2.4. Inflammatory Profile by Alleles of the UCP2 rs659366 Polymorphism

Table 4 presents the inflammatory characteristics according to *UCP2* alleles, showing that the platelet-to-lymphocyte ratio (PLR) was higher among AA genotype carriers (*p* = 0.036). No significant differences were observed for the other parameters.

### 2.5. Interaction Between UCP2 rs659366 Polymorphism, Dietary Capsaicin Intake and NLR

**Figure 1:**
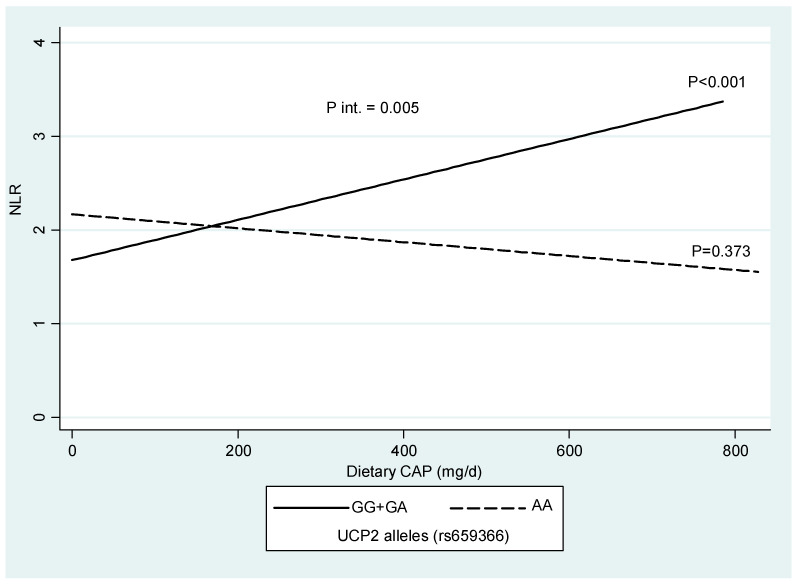
Interaction plot concerning the *UCP2* rs659366 polymorphism and dietary CAP intake concerning the inflammatory NLR marker. *p* value is adjusted by age, sex, BMI, and total energy intake.

A significant interaction (*p* = 0.005) was identified between CAP consumption and the *UCP2* rs659366 polymorphism (Figure 1). Among subjects with the G allele, higher CAP consumption was associated with a greater NLR index (*p* < 0.001). No other significant interactions were found between CAP consumption and *UCP2* genotypes for other inflammatory markers, including PLR (*p* = 0.634), EBR (*p* = 0.711), ELR (*p* = 0.491), and LMR (*p* = 0.689).

## 3. Discussion

In this study, the frequencies of the *UCP2* rs659366 polymorphism in participating patients from Tijuana were 40.2% for the ancestral allele G and 59.8% for the risk allele A. The genotype frequencies were 3.7% for homozygous GG, 73% for heterozygous GA, and 23.3% for homozygous AA. In the Mexican population from Mexico City and its surroundings, the frequency of the G allele was 57.1% and 42.9% for the A allele in cases of premature coronary artery disease (PCAD). The frequencies for the GG, GA, and AA genotypes were 31.5%, 51.1%, and 14.85%, respectively [19]. Internationally, our results are also comparable with the frequencies of this genetic variant in the Latin American population [20,21]. In a Brazilian population, the study of this polymorphism found an allele frequency of 40.5% for the G allele and 59.8% for the A allele in both diabetic and non-diabetic individuals, similar to the results found in this study [22].

Polymorphisms in the *UCP2* gene may influence the expression and function of the UCP2 protein. These changes can directly affect the organism’s ability to regulate energy homeostasis, oxidative stress, and inflammation [23,24,25]. In our results, approximately 67% of the patients were overweight or obese according to BMI, and it was observed that individuals with the risk allele A had a higher HC compared to those with the ancestral allele G. In the Indonesian population, the *UCP2* rs660339 polymorphism was also associated with obesity and body fat distribution [26]. In a previous study of a Brazilian population, the *UCP2* rs660339 AA genotype was associated with higher BMI [27]. Moreover, it was demonstrated that administration of CAP (10 mg/kg/d) promoted energy metabolism and suppressed body fat accumulation in mice, suggesting that this positive regulation appears to mediate the effects of CAP on metabolism [28].

In our results, no significant differences in the lipid profile of the studied subjects were found, suggesting that other factors may influence lipid outcomes. Notably, the effects of CAP on lipid catabolism in differentiated 3T3 adipocytes were investigated using CAP concentrations of 0.1 mM, 1 mM, and 10 mM for 24 h. It was demonstrated that the genes hormone-sensitive lipase (*HSL*), carnitine palmitoyltransferase I α (*CPTI-α*), and *UCP2* were significantly regulated and that these genes are involved in lipid catabolism [29,30]. These results suggest that CAP affects lipolysis through lipid catabolism, including thermogenesis via UCP2 [31].

Metabolic health has attracted significant interest in recent years, and the *UCP2* rs659366 polymorphism has emerged as a key factor in glucose metabolism and cardiovascular risk. Our results indicate an increase in glucose concentration in patients with the *UCP2* rs659366 risk allele A. In a diabetic population, individuals with the A allele of the *UCP2* rs659366 polymorphism had a significantly lower risk of developing coronary disease, suggesting a protective effect of this variant on cardiovascular risk in diabetics [32]. Additionally, increased expression of *UCP2* induced by TRPV1 exerted a protective antioxidant effect on the liver in non-alcoholic fatty liver disease and on vascular endothelium during hyperglycemia [18]. These studies highlight the importance of understanding how genetic factors interact with metabolism and cardiovascular health in different populations.

CAP has potential anti-inflammatory effects, but polymorphisms in the *UCP2* gene may modify the body’s response to its consumption, which could have significant implications for metabolic and inflammatory health [15,17]. In a study where CAP supplementation (doses between 0.5 and 50 µM) was administered to adipocyte cells in culture, it was found to inhibit adiposity-dependent inflammation, reduce the release of pro-inflammatory cytokines, and decrease leptin levels. This demonstrated that adipocyte differentiation and the expression of inflammatory proteins related to adipose tissue were suppressed with dose-dependent CAP supplementation [33].

The positive regulation of UCP2 appears to mediate the effects of CAP on metabolism [28]. Genetic polymorphisms may cause some individuals to respond better than others to the beneficial effects of CAP. In this study, we found a higher PLR for the A allele of the *UCP2* rs659366 polymorphism compared to the G allele. In this context, it was investigated whether supplementation with 150 mg CAP affected autonomic and cardiac electrophysiological activity during exercise in obese individuals in Japan, and how polymorphisms of the *UCP2* and *UCP3* genes may influence this response. The results indicated that CAP activated the expression of *UCP2* and reduced inflammation, although its effectiveness depends directly on the genetic variants present in the individual [34].

CAP interacts differently in individuals with the *UCP2* polymorphism, which may predispose some to chronic inflammation. Fat storage in obesity can lead to insulin resistance and stimulate a chronic, subacute inflammatory state. In our study, we found that in subjects with the G allele of the *UCP2* rs659366 variant, higher CAP consumption was associated with a higher NLR. A study reported an association between high-sensitivity C-reactive protein (a biomarker of inflammation) and the *UCP2* polymorphism, suggesting the role of UCP2 in the inflammatory response [25]. Neutrophils and lymphocytes are cells of the first line of defense against infections and play a role in inflammation. Thus, NLR is a promising new inflammatory biomarker that is low-cost and reliable for assessing the inflammatory status and prognosis of various diseases such as cardiovascular diseases, diabetes mellitus, obesity, and cancer, among others [35,36].

Recent research has revealed important insights into how CAP can positively influence endothelial cell function under hyperglycemic conditions [12]. When endothelial cells were exposed to high glucose levels (30 mmol/L), researchers observed a significant reduction in TRPV1 expression and PKA phosphorylation. This finding highlights the complex role of NADPH in generating reactive oxygen species (ROS) within this signaling framework. Notably, higher glucose levels led to overexpression of the p22 subunit of NADPH oxidase, a response that CAP treatment effectively countered. CAP reduced ROS production while simultaneously enhancing nitric oxide (NO) synthesis, demonstrating its potential as a protective agent against oxidative stress. This suggests that CAP activates TRPV1, which helps reduce ROS levels and increases NO production through the PKA/UCP2 signaling pathway. Moreover, administering CAP at a concentration of 1 mmol/L significantly increased TRPV1 expression in endothelial cells, resulting in a notable increase in UCP2 levels. This enhancement suggests that the PKA phosphorylation pathway is activated through TRPV1 signaling, further underscoring the therapeutic potential of CAP in combating endothelial dysfunction caused by hyperglycemia [12]. In a related study on sepsis induced by lipopolysaccharides (LPS), treatment with CAP was found to increase protein levels of caspase-1, ASC, and NLRP3, as well as the number of PI-positive cells, indicating activation of pyroptosis and apoptosis [37]. However, CAP showed a remarkable ability to inhibit both pyroptosis and apoptosis triggered by LPS. It protected against mitochondrial dysfunction through the TRPV1/UCP2 pathway, effectively reducing cell apoptosis by lowering levels of cleaved caspase-1, ASC, and NLRP3. This indicates a significant reduction in pyroptosis, particularly through CAP’s inhibitory effect on LPS-induced expression of the inflammatory cytokines IL-1β and IL-18, which play crucial roles in regulating inflammatory responses [37].

The results of this research are important for expanding current knowledge about CAP consumption and its effects on health in obesity and inflammation. In particular, this study suggests that the *UCP2* rs659366 genotype influences the impact of dietary CAP on inflammation status, where individuals with the G allele (GG + GA), but not AA homozygotes, had greater inflammation scores (based on NLR) as they increased CAP intake. Hence, this finding supports the need to personalise CAP-based nutritional recommendations based on *UCP2* profiles (limiting CAP in G allele carriers), especially when there is an underlying inflammatory process, which may help optimise the treatment of this condition under a precision nutrition approach [38]. Nonetheless, additional studies are required to validate these results and translate them into clinical practice. Furthermore, this knowledge could help explain some inconsistencies in epidemiological studies that have investigated the association of CAP with obesity and metabolic disorders.

The main contribution of this study is the analysis of the interaction between the *UCP2* gene, CAP intake, and inflammation, which has not been previously explored in the Mexican population. A limitation of this study is that there may be some bias in the self-reporting of CAP consumption, in addition to the cross-sectional design. However, the use of a validated and specific questionnaire to estimate CAP intake in the Mexican population could help reduce this inherent bias [39,40]. Nevertheless, longitudinal studies tracking CAP intake over extended periods and clinical trials with varying CAP dosages are still needed to further investigate the effects of CAP consumption on inflammation and metabolic health features based on genetic backgrounds and to explore causality. Moreover, since relationships between CAP intake, UCP2 polymorphism, and inflammation were found in volunteers living in northern Mexico with particular genetic and environmental interactions, these associations should also be assessed in other regions of the country, as well as in other populations worldwide with varying CAP exposure.

In conclusion, this study suggests that patients with the G allele of the *UCP2* rs659366 polymorphism exhibit greater inflammatory status when they consume higher amounts of dietary CAP, supporting the idea that the prescription of this compound should be personalised based on individual genetic backgrounds.

## 4. Materials and Methods

### 4.1. Study Design and Participants

In this cross-sectional, analytical study, 212 healthy adult patients (self-reported) of both sexes, aged 18–65 years, who were residents of the city of Tijuana, Baja California, Mexico, were randomly recruited through non-probabilistic sampling. Exclusion criteria included patients with thyroid disorders or metabolic diseases requiring pharmacological treatment; subjects on a special diet or calorie/macronutrient-restricted diets in the three months prior to the study; subjects taking any medication that could affect taste perception or biochemical blood levels; patients who consumed alcohol; and those with hypersensitivity to chili or CAP.

This study was reviewed and approved by the Research Ethics Committee of the Autonomous University of Baja California (UABC), with approval code: D235 (accepted on 22 October 2019). The updated version of the ethical principles for research involving human beings from the 2013 Declaration of Helsinki was considered for this research [41]. Once participants were informed about the purposes of the study, they voluntarily signed written informed consent to be included in this protocol. The confidentiality of all patient data (including demographic, anthropometric, biochemical, and genetic information) was guaranteed through the use of codes in the patient records. The information was used strictly for research purposes, and only registered researchers had access to the database. Patient DNA was stored in a DNA library under the protection of the UABC.

### 4.2. Anthropometric Measurements and Arterial Measurement

To estimate the height of the patients, a stadiometer (Rochester Clinical Research, New York, NY, USA) was used. To measure body mass index (BMI) and body fat, a bioimpedance device (Tanita SC-331S body composition analyser, Tanita Corporation, Tokyo, Japan) was used. BMI was calculated by dividing body weight by height in square meters (kg/m^2^). An ergonomic measuring tape (SECA, Wandsbek, Hamburg, Germany) was used to measure waist circumference (WC) and hip circumference (HC). Trained nutritionists were responsible for taking the measurements of WC and HC following the procedures. Blood pressure was measured using an automatic digital arm blood pressure monitor (Omron HEM-7120, OMRON Corporation, Kyoto, Japan). The waist-to-hip ratio (WHR) was calculated by dividing WC by HC.

### 4.3. Dietary Intake and Appetite

To determine the average daily intake of macronutrients and micronutrients, three food records (two weekdays and one weekend day) were collected and analysed using Nutritionist Pro software version 8.1 (Axxya Systems, Stafford, TX, USA). To assess CAP consumption, a validated semi-quantitative food frequency questionnaire (FFQ) was used, which included the most commonly consumed types of peppers and dishes prepared with chili in Mexico [39,40]. Participants were asked how frequently they had consumed chili over the past three months, with response categories of never, monthly, weekly, and daily. The FFQ included standardised quantitative portions of chili to determine the portion size consumed, expressed in household measures or grams. The frequency of chili consumption for the predefined portions of chilies and dishes prepared with chilies was multiplied by the CAP content of each chili, depending on whether it was fresh or dried. To calculate the total intake of CAP for each participant, the CAP content from the daily reported consumption of each chili and chili dish was summed [39,40].

Appetite was assessed using the Simplified Nutritional Appetite Questionnaire (SNAQ), a four-item tool that includes self-assessments of appetite, satiety, food taste, and number of meals per day [42]. The total score was used as a continuous variable, ranging from 4 to 20.

### 4.4. Laboratory Tests

The patients were scheduled for a 12-h fasting period before the collection of a 10 mL blood sample via venepuncture. Three milliliters of blood were placed in tubes containing K2 EDTA anticoagulant, and 7 milliliters were placed in a red tube without additives. These samples were centrifuged at 3000 rpm for 10 min to obtain serum, and the biochemical tests were performed immediately. The biochemical tests included fasting glucose, total cholesterol, triglycerides, high-density lipoprotein cholesterol (HDL-c), and a liver profile, all determined using commercial kits and analysed on the Mindray BS-200 equipment (Mindray Medical International Limited, Shenzhen, China). Reagents, controls, and calibrators from Pointe Scientific were used. Low-density lipoprotein cholesterol (LDL-c) was calculated using the Friedewald equation. Non-high-density lipoprotein cholesterol (non-HDL-c) was estimated as TC minus HDL-c. The triglyceride to glucose (TyG) index, reflecting insulin resistance [43], was calculated as follows: ln (fasting triglycerides (mg/dL) × fasting glucose (mg/dL)/2). Inflammatory indices were calculated using simple ratios between parameters with absolute values: neutrophil-to-lymphocyte ratio (NLR), platelet-to-lymphocyte ratio (PLR), eosinophil-to-lymphocyte ratio (ELR), and lymphocyte-to-monocyte ratio (LMR) [44,45].

### 4.5. UCP2 Genotyping

Mononuclear cells from peripheral blood (PBMCs) were isolated from the patients using the Lymphoprep method (Serumwerk Bernburg AG, Bernburg (Saale), Germany). They were stored at −80 °C for later use. Genomic DNA was extracted from the PBMCs using the Wizard^®^ Genomic DNA Purification Kit (PROMEGA Corporation, Madison, WI, USA), and DNA quantification was performed by spectrophotometry. The quantity, quality, and purity of the DNA were assessed. A 1% agarose gel electrophoresis stained with SYBR GREEN was performed to evaluate DNA integrity. Genotyping of the rs659366 polymorphism (*UCP2*) was conducted using real-time PCR on a 7300 Real-Time PCR System (Applied Biosystems, Foster City, CA, USA) with TaqMan^®^ SNP genotyping assays (catalogue number C___8760350_10 C; ThermoFisher Scientific, Waltham, MA, USA). The sequence used was: TGACCCGTCCTGTGGGGGTAACTGA[C/T]GCGTGAACAGCCAACAATTGGGCCC. The reaction conditions followed the supplier’s instructions. Positive controls were included to verify the three possible genotypes (GG, GA, and AA) on each 96-well plate, and 20% of the samples were reprocessed (all of which were 100% concordant), as well as negative controls on each 96-well plate.

### 4.6. Statistical Analysis

The Kolmogorov–Smirnov test was performed to assess the normality of the main study variables (CAP intake and NLR). Continuous variables were expressed as means ± standard deviations, while categorical variables were presented as frequencies and percentages. For the comparison of *UCP2* genotypes, participants were grouped as G carriers (genotypes GG + GA) and homozygotes for the risk allele A (AA genotype). Comparisons of genotypes for continuous and categorical variables were performed using Student’s *t* tests and Chi-square tests, respectively. To investigate relevant interactions between genes and diet, multiple linear regression analyses were conducted, adjusted for energy intake, age, sex, and BMI. Statistical analyses were performed using Stata 12 statistical software (StataCorp LLC, College Station, TX, USA; www.stata.com, accessed on 21 February 2025) and IBM SPSS Statistics version 21.0 (IBM Inc., Armonk, NY, USA). Statistical significance was set at two-tailed *p* < 0.05.

## 5. Conclusions

In conclusion, patients with the G allele of the *UCP2* rs659366 polymorphism show increased inflammation as they consume more CAP in their diet. This finding establishes a basis for the personalised recommendation of CAP for the precise management of inflammation.

## Figures and Tables

**Table 1:** Demographic, anthropometric, and clinical characteristics according to genotypes of the *UCP2* rs659366 polymorphism.

Variables	GG + GA *n* = 166	AA *n* = 46	*p* Value
Age (years)	37.84 ± 12.7	37.15 ± 11.1	0.741
Sex (F/M)	102/64	27/19	0.431
Normal weight, *n* (%)	51 (30.7)	8 (17.4)	0.088
Overweight, *n* (%)	60 (36.1)	16 (34.7)	
Obesity, *n* (%)	53 (31.9)	22 (47.8)	
BMI (kg/m^2^)	26.9 ± 4.4	29.0 ± 1.4	0.557
Total body fat (%)	34.7 ± 9.0	35.9 ± 9.3	0.588
WC (cm)	89.6 ± 14.6	93.0 ± 13.9	0.155
HC (cm)	104.8 ± 10.6	108.5 ± 10.1	**0.040**
WHR	0.85 ± 0.05	0.85 ± 0.09	0.992
SBP (mmHg)	121.1 ± 15.8	119.1 ± 24.8	0.551
DBP (mmHg)	78.6 ± 9.7	78.5 ± 10.8	0.962

Values are presented as means ± standard deviations. Sex is represented as number of cases. Abbreviations: F: female; M: male; BMI: body mass index; WC: waist circumference; HC: hip circumference; WHR: waist to hip ratio; SBP: systolic blood pressure; DBP: diastolic blood pressure. The statistical analyses for continuous variables were screened using Student’s *t* tests and the Chi-Square test was used for assessment of categorical variables. Bold numbers indicate *p* < 0.05.

**Table 2:** Capsaicin intake by genotypes of the *UCP2* rs659366 polymorphism.

Variables	GG + GA *n* = 166	AA *n* = 46	*p* Value
CAP (mg/d)	666.4 ± 6699.4	162.2 ± 195.6	0.615
Appetite score	11.3 ± 2.7	11.4 ± 2.7	0.908
Total calories/d	2018.1 ± 685.3	2149.4 ± 941.9	0.329
Total proteins (%)	20.2 ± 15.7	18.8 ± 4.0	0.581
Total fat (%)	37.7 ± 7.8	37.4 ± 9.5	0.823
Total carbohydrates (%)	42.2 ± 9.6	43.3 ± 9.7	0.560

Values are presented as means ± standard deviations. CAP: capsaicin. Statistical analyses were performed using Student’s *t* tests.

**Table 3:** Biochemical profile by genotypes of the *UCP2* rs659366 polymorphism.

Variable	GG + GA *n* = 166	AA *n* = 46	*p* Value
Fasting glucose (mg/dL)	93.8 ± 12.6	99.19 ± 17.5	**0.020**
Total cholesterol (mg/dL)	192.7 ± 38.9	186.4 ± 32.4	0.321
HDL-c (mg/dL)	45.97 ± 13.1	42.5 ± 10.8	0.101
Non-HDL-c (mg/dL)	146.7 ± 37.9	143.9 ± 34.13	0.656
LDL-c (mg/dL)	125.6 ± 35.2	122.7 ± 27.3	0.608
Triglycerides (mg/dL)	105.1 ± 59.7	115.5 ± 69.2	0.315
TyG	4.52 ± 0.28	4.58 ± 0.30	0.182
Uric acid (mg/dL)	5.4 ± 1.8	5.7 ± 1.9	0.247
Total proteins (g/dL)	7.48 ± 0.4	7.3 ± 0.5	**0.039**
Albumin (g/dL)	4.0 ± 0.1	3.9 ± 0.1	0.112
Globulins	3.4 ± 0.4	3.3 ± 0.5	0.441
LDH (U/L)	213.5 ± 123.4	223.3 ± 115.5	0.627
AST (U/L)	38.3 ± 20.8	37.6 ± 17.7	0.845
ALT (U/L)	29.4 ± 32.1	31.3 ± 23.8	0.700
GGT (U/L)	18.4 ± 13.0	21.0 ± 17.7	0.276
TB (umol/L)	1.2 ± 0.5	0.9 ± 0.3	**0.001**
DB (umol/L)	0.17 ± 0.08	0.14 ± 0.10	0.128
ALP (U/L)	161.0 ± 64.1	148.0 ± 58.7	0.218
Urea (mg/dL)	24.9 ± 13.2	22.5 ± 12.1	0.266
Creatinine (mg/dL)	0.66 ± 0.18	0.75 ± 0.20	**0.006**
Creatine kinase	234.8 ± 234.5	396.8 ± 779.1	**0.035**

Values are presented as means ± standard deviations. Abbreviations: HDL-c: high-density lipoprotein cholesterol; LDL-c: low-density lipoprotein cholesterol; LDH: lactic dehydrogenase; AST: aspartate aminotransferase; ALT: alanine aminotransferase; GGT: gamma glutamyl transpeptidase; TB: total bilirubin; DB: direct bilirubin; ALP: alkaline phosphatase; TyG: triglyceride and glucose index. Statistical analyses were performed using Student’s *t* tests. Bold numbers indicate *p* < 0.05.

**Table 4:** Inflammatory characteristics by genotypes of the *UCP2* rs659366 polymorphism.

Variable	GG + GA *n* = 166	AA *n* = 46	*p* Value
NLR	1.96 ± 1.10	2.04 ± 1.09	0.665
PLR	133.89 ± 54.48	154.44 ± 73.06	**0.036**
EBR	4.84 ± 9.77	5.31 ± 8.30	0.762
ELR	0.091 ± 0.08	0.08 ± 0.07	0.635
LMR	6.08 ± 2.43	5.847 ± 2.33	0.549

Values are presented as means ± standard deviations. Abbreviations: NLR: neutrophil-to-lymphocyte ratio; PLR: platelet-to-lymphocyte ratio; EBR: eosinophil-to-basophil ratio; ELR: eosinophil-to-lymphocyte ratio; LMR: lymphocyte-to-monocyte ratio. Statistical analyses were performed using Student’s *t* tests. Bold numbers indicate *p* < 0.05.

## Data Availability

The original contributions presented in this study are included in the article. Further inquiries can be directed to the corresponding author(s).
